# The influence of temperature and genomic variation on intracranial EEG measures in people with epilepsy

**DOI:** 10.1093/braincomms/fcae269

**Published:** 2024-09-10

**Authors:** Olivia C McNicholas, Diego Jiménez-Jiménez, Joana F A Oliveira, Lauren Ferguson, Ravishankara Bellampalli, Charlotte McLaughlin, Fahmida Amin Chowdhury, Helena Martins Custodio, Patrick Moloney, Anna Mavrogianni, Beate Diehl, Sanjay M Sisodiya

**Affiliations:** Sir Jules Thorn Telemetry Unit, National Hospital for Neurology and Neurosurgery, London WC1N 3BG, UK; Department of Clinical and Experimental Epilepsy, UCL Queen Square Institute of Neurology, London WC1N 3BG, UK; Chalfont Centre for Epilepsy, Buckinghamshire SL9 0RJ, UK; Sir Jules Thorn Telemetry Unit, National Hospital for Neurology and Neurosurgery, London WC1N 3BG, UK; Department of Clinical and Experimental Epilepsy, UCL Queen Square Institute of Neurology, London WC1N 3BG, UK; Institute for Environmental Design and Engineering, The Bartlett School of Environment, Energy and Resources, University College London, London WC1H 0NN, UK; Department for Environmental Health, Harvard T.H. Chan School of Public Health, Boston, MA 02115, USA; Department of Clinical and Experimental Epilepsy, UCL Queen Square Institute of Neurology, London WC1N 3BG, UK; Chalfont Centre for Epilepsy, Buckinghamshire SL9 0RJ, UK; Sir Jules Thorn Telemetry Unit, National Hospital for Neurology and Neurosurgery, London WC1N 3BG, UK; Sir Jules Thorn Telemetry Unit, National Hospital for Neurology and Neurosurgery, London WC1N 3BG, UK; Department of Clinical and Experimental Epilepsy, UCL Queen Square Institute of Neurology, London WC1N 3BG, UK; Department of Clinical and Experimental Epilepsy, UCL Queen Square Institute of Neurology, London WC1N 3BG, UK; Chalfont Centre for Epilepsy, Buckinghamshire SL9 0RJ, UK; Department of Clinical and Experimental Epilepsy, UCL Queen Square Institute of Neurology, London WC1N 3BG, UK; Chalfont Centre for Epilepsy, Buckinghamshire SL9 0RJ, UK; Institute for Environmental Design and Engineering, The Bartlett School of Environment, Energy and Resources, University College London, London WC1H 0NN, UK; Sir Jules Thorn Telemetry Unit, National Hospital for Neurology and Neurosurgery, London WC1N 3BG, UK; Department of Clinical and Experimental Epilepsy, UCL Queen Square Institute of Neurology, London WC1N 3BG, UK; Department of Clinical and Experimental Epilepsy, UCL Queen Square Institute of Neurology, London WC1N 3BG, UK; Chalfont Centre for Epilepsy, Buckinghamshire SL9 0RJ, UK

**Keywords:** epilepsy, EEG, seizures, climate change, heatwaves

## Abstract

Heatwaves have serious impacts on human health and constitute a key health concern from anthropogenic climate change. People have different individual tolerance for heatwaves or unaccustomed temperatures. Those with epilepsy may be particularly affected by temperature as the electroclinical hallmarks of brain excitability in epilepsy (inter-ictal epileptiform discharges and seizures) are influenced by a range of physiological and non-physiological conditions. Heatwaves are becoming more common and may affect brain excitability. Leveraging spontaneous heatwaves during periods of intracranial EEG recording in participants with epilepsy in a non–air-conditioned telemetry unit at the National Hospital for Neurology and Neurosurgery in London from May to August 2015–22, we examined the impact of heatwaves on brain excitability. In London, a heatwave is defined as three or more consecutive days with daily maximum temperatures ≥28°C. For each participant, we counted inter-ictal epileptiform discharges using four 10-min segments within, and outside of, heatwaves during periods of intracranial EEG recording. Additionally, we counted all clinical and subclinical seizures within, and outside of, heatwaves. We searched for causal rare genetic variants and calculated the epilepsy PRS. Nine participants were included in the study (six men, three women), median age 30 years (range 24–39). During heatwaves, there was a significant increase in the number of inter-ictal epileptiform discharges in three participants. Five participants had more seizures during the heatwave period, and as a group, there were significantly more seizures during the heatwaves. Genetic data, available for eight participants, showed none had known rare, genetically-determined epilepsies, whilst all had high polygenic risk scores for epilepsy. For some people with epilepsy, and not just those with known, rare, temperature-sensitive epilepsies, there is an association between heatwaves and increased brain excitability. These preliminary data require further validation and exploration, as they raise concerns about the impact of heatwaves directly on brain health.

## Teaser

McNicholas *et al*. evaluated electroclinical markers in nine participants with epilepsy who experienced coincidental heatwaves during inpatient intracranial EEG monitoring from 2015 to 2022. In three participants, there were significantly more inter-ictal epileptiform discharges during heatwaves compared to non-heatwaves. As a group, there was a significant increase in seizure numbers during heatwaves.

## Graphical Abstract

**Graphical Abstract:**
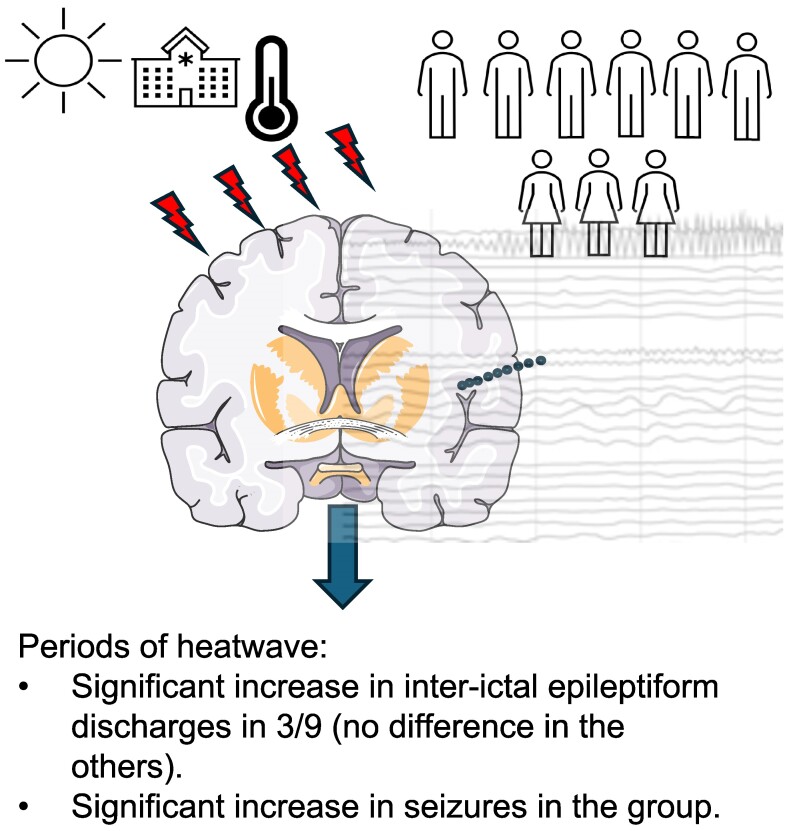

## Introduction

Climate change is happening around us: 2023 was the hottest year on record. Climate change will lead to increasing temperatures and more severe and frequent periods of extreme heat, called heatwaves.^1^ A heatwave is defined as a period of at least three consecutive days of unusually hot weather, with a daily maximum above a specified threshold.^2^ Climate change will have major consequences for human health.^3^ Whilst there have been warnings about the impacts on human health generally, and for ‘vulnerable populations’, studies on the effects of climate change on specific diseases have been limited, with little granularity expanding on the very broad term ‘vulnerable’. Neurological diseases are burdensome in general, and epilepsy is amongst the most burdensome, as one of the most common chronic neurological conditions, affecting over 50 million people worldwide.^4^ Epilepsy may also provide insights into brain health, and response to climate change, more generally. Some seizure types (e.g. febrile seizures) are more likely to happen with elevated body temperature, often in combination with a systemic inflammatory response. Some epilepsies, typically rare ‘monogenic’ conditions such as Dravet syndrome and *CHD2*-related epilepsy, feature seizures that can be triggered by high ambient temperatures, or rapid change in ambient temperature.^5^ Temperature elevation may provoke seizures in a broad range of epilepsies.^6^ A study from Brazil reported a 4.3% increase in the risk of hospitalization for seizures with each 1°C increase in external temperature.^7^ Inter-ictal epileptiform discharges (IEDs) are a marker of epileptogenic tissue in the brain.^8^ IEDs can vary in frequency according to epilepsy syndrome, different physiological and non-physiological conditions including during sleep, with varying hormone levels and with anti-seizure medication (ASM) treatment.^9,10^ A study performed during intraoperative electrocorticography in children showed that cortical irrigation with 150 cm^3^ chilled (4°C) normal saline solution reduced the average number of IEDs.^11^ Ion channels, whose activity is critical to brain function and whose involvement is central to seizures, demonstrate exquisite sensitivity to ambient temperature; mutated ion channels may be the cause of some epilepsies, and mutant channel function typically retains steep dependence on ambient temperature.^12,13^ These observations suggest that brain function, in both healthy people and those with epilepsy, may show sensitivity to unaccustomed high or low ambient temperature, and raise concerns about brain function as climate change progresses, especially during associated heatwaves. We leveraged existing data sets of intracranial EEG (icEEG) recordings undertaken in people with medication-resistant focal epilepsy that encompassed heatwaves on a non-temperature–controlled video EEG monitoring unit. Our objective was to test the hypothesis that heatwaves increase brain cortical excitability, potentially aggravating specific genetic factors underpinning epilepsy. IcEEG studies are ideal for such work as they are typically long studies, enabling comparison between heatwave and non-heatwave settings in the same person under stable conditions.

## Materials and methods

We selected all participants who, as part of their ongoing assessment for epilepsy surgery, underwent icEEG, sampling mesial temporal and deep structures, around heatwaves from May to August 2015–22, and whose recording included both non-heatwave periods and the entire duration of a heatwave during the same admission. We excluded cases with only neocortical sampling, i.e. grids only.

### Ethics

All individuals included had given written informed consent to a study of genomic influences on epilepsy (Research Ethics Committee identifier: 11/LO/2016). Additionally, this study was undertaken as part of an independently approved (Clinical Audit and Quality Improvement Subcommittee, Queen Square Division, University College London Hospitals NHS Foundation Trust; registration number: 62-202122-SE) service evaluation on environments at our hospital with respect to the Greener NHS programme.^14^

### Genomic analysis

Both rare and common genomic variation can contribute to epilepsy. To test the hypothesis that different neurophysiological responses to ambient temperatures might be influenced by genetic variation, we examined both rare (potentially causal) variants and common variants [through polygenic risk scores (PRSs)]. DNA was available for eight study participants and was used to obtain whole-genome sequencing (WGS). Data were processed as previously described,^15^ with further details in the Supplementary material.

PRS for epilepsy was calculated in eight of the nine participants in this study, GEL Epilepsy and GEL Control cohorts (see Supplementary Figs 1 and 2). On genetic analysis, one participant (#9) was of non-European ancestry, but was kept in this analysis for this pilot study given the small number of participants overall, and that this study was not intended to explore PRS differences between cohorts (see Supplementary Fig. 3).

### Electrophysiological recording setting

**Figure 1:**
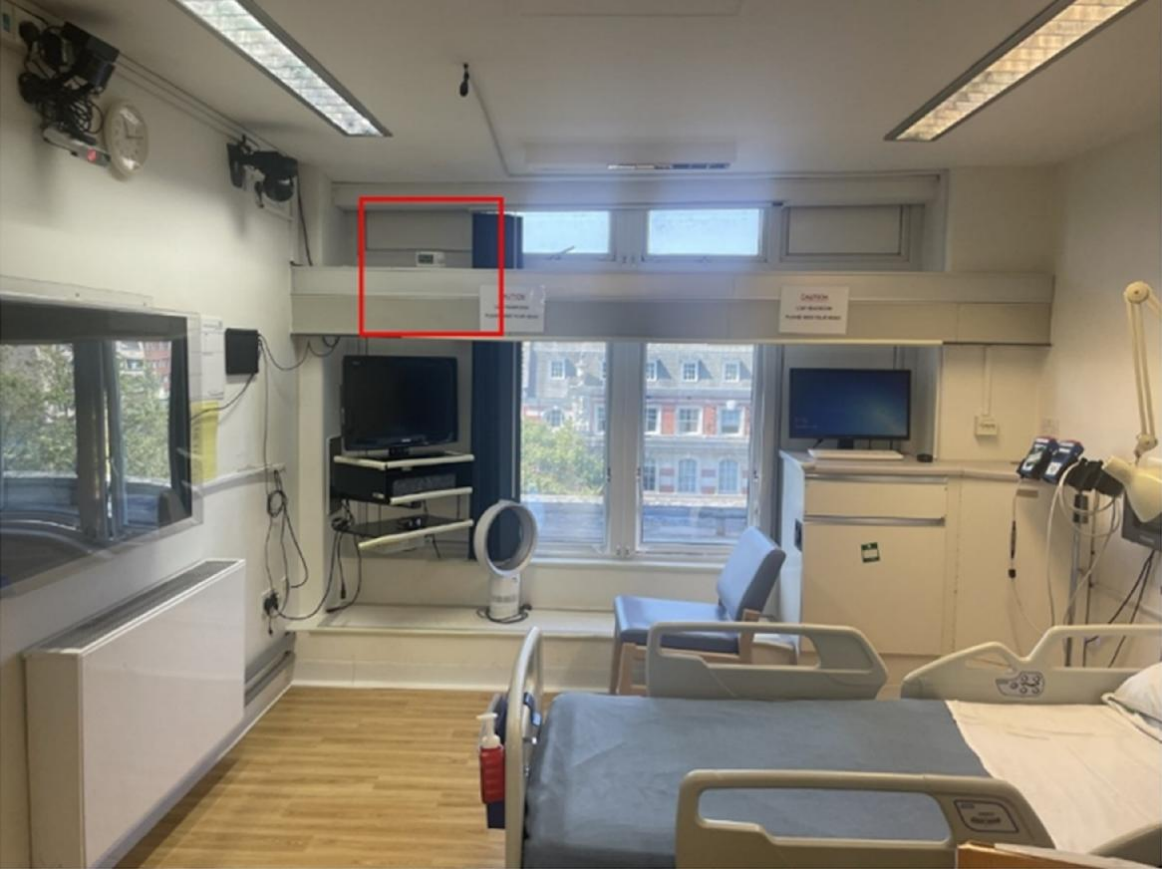
**Study setting.** An example of an icEEG video-telemetry room, with west-facing window, at the NHNN. All rooms used for icEEG are west-facing, with large windows, which cannot fully open due to legal reasons, and have no air conditioning. The position of the Onset HOBO data logger (dry bulb temperature recorder) is outlined with the square.

IcEEG data were recorded in the National Hospital for Neurology and Neurosurgery (NHNN) telemetry unit, a west-facing environment that is not air-conditioned (Fig. 1): it is situated on the eastern edge of Queen Square, London, UK, and its concrete structure was built in 1859.

#### Non-heatwave and heatwave characterization

A non-heatwave period was defined as a minimum of 3 days during which the daily maximum external temperature was below 28°C, whereas a heatwave period was defined as at least three consecutive days with daily maximum external temperatures exceeding 28°C (28°C is the local threshold for London, UK).^16^ Where possible, non-heatwave days were sampled at least 48 h before or after the heatwave to attempt to account for potential thermal mass effects in the telemetry unit, where the building may retain heat for up to several hours after a heatwave. Heatwave and non-heatwave days were selected without knowledge of the number of seizures on any day during the icEEG recording.

Temperature data were acquired as follows. Indoor measurements of dry bulb temperature were collected between April 2022 and August 2023 at sub-hourly time intervals inside the icEEG recording room using a HOBO UX100-001 USB Temperature Data Logger (range −20 to 70°C; accuracy 0.21°C from 0 to 50°C; Onset Computer Corporation, MA, USA). The HOBO data logger was placed on a high beam out of direct sunlight and away from sources of heat (Fig. 1). The time-series plot of the indoor and outdoor temperature data aggregated to daily means can be viewed in Supplementary Fig. 4. Prior to 29 April 2022, no measurements of indoor temperature were available from the telemetry unit. A statistical approach was therefore used to model indoor temperatures on icEEG study dates when direct measurements were not available.

#### Indoor temperature model

The indoor temperature measurements were linked with outdoor meteorological data from the Visual Crossing Weather API at St. James Park (London, UK) monitoring station, the closest outdoor monitor to NHNN, to extrapolate indoor temperature readings for dates prior to 29 April 2022.^17^ All data processing and analyses were carried out in RStudio (RStudio, 2020). Further data analysis is available in the Supplementary material.

**Figure 2:**
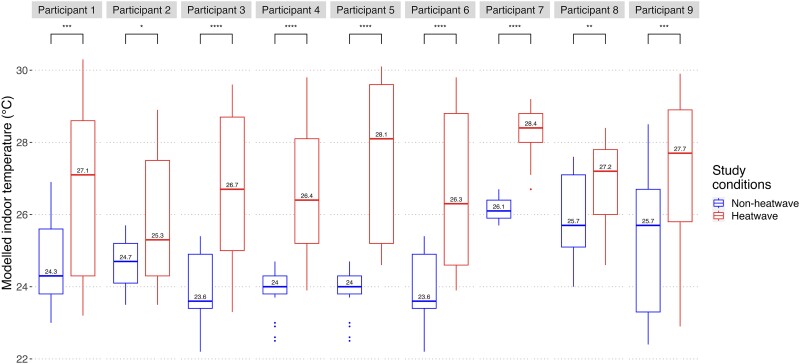
**Modelled indoor temperatures on heatwave and non-heatwave days.** The distribution of modelled indoor temperatures on heatwave and non-heatwave days during each participant’s icEEG study, shown with the *P*-value between non-heatwave and heatwave days for each participant. *P-*values were attained via an unpaired *t*-test. *P < 0.05; **P < 0.01; ***P < 0.001; ****P <0.0001. The boxplots encompass data points for indoor temperature estimates (in °C) in the telemetry unit at hourly time intervals, aggregated by heatwave and non-heatwave days for each participant. The central line in each boxplot represents the median, whilst the box shows the interquartile range and the lower and upper whiskers show the minimum and maximum for each group, respectively. Note that *modelled* temperature estimates are shown for all participants except participant #7, for whom directly recorded indoor measurements were available and are shown.

A random forest model was used to predict study date indoor conditions from the outdoor meteorological data when indoor measurement data were not available (see Supplementary Figs 4–6 and Supplementary Table 1 for further details, including model performance), implemented in RStudio using the package *Ranger.*^18^ Figure 2 shows the distribution of modelled indoor temperatures for each participant during heatwave and non-heatwave epochs.

### EEG recording

Only pre-existing data were used. The icEEG data were recorded using a SD LTM 64 Express, Micromed amplifier (Micromed, Treviso, Italy), with a sampling rate of 1024 Hz and digital filters of 0.008–400 Hz bandpass. Implantation of electrodes was driven clinically and depended on the hypothesized epileptogenic areas as determined by the multidisciplinary team considering semiology, previous video-telemetry findings on scalp EEG and imaging data. The anatomical location of each electrode contact was verified on a post-implantation MRI brain scan. We sampled only from the electrode contacts which were implanted in the anatomical region of interest.

### IED identification

EEG data were reviewed using a bipolar montage, 400 µV/cm sensitivity and a 15-s time base. The EEG was first visually screened to discard artefacts and remove faulty channels or electrodes not in contact with the brain. The following criteria for EEG segment selection were used: awake, resting recording, more than 24 h after implantation to account for anaesthetic effects, the same ASM doses during the selected heatwave and non-heatwave days, at least an hour before or after a clinical seizure and not during or within the hour after periods of additional intervention, e.g. cortical stimulation and single-pulse electrical stimulation.^19-22^ Awake EEG data were examined by two independent EEG readers (O.C.M. and B.D.); reviewers were blinded to whether the EEG was from non-heatwave and heatwave epochs.

For each participant, we selected four 10-min epochs (i.e. 40 min in total) of icEEG during the daytime (between 12:00 and 19:00), and on the hour, during a selected non-heatwave and heatwave day. If we were unable to sample from the 10-min epoch on the hour (e.g. participant asleep), then the epoch sampling was moved forward to the next hour. The same 10-min epochs were used for both non-heatwave and heatwave epochs. We used 10-min epochs rather than 24-h epochs due to the burden of manual IED counting.

All IEDs were annotated and counted based on visual analysis only. There is no generally accepted definition of IEDs in icEEG: only graphoelements that stood out from the background, had a rapid upslope, were of short duration and had an aftergoing slow wave were marked as an IED.^21,22^

We analysed IEDs in the depth electrode contact locations which had been implanted across many of the participants. These contacts sampled from the amygdala; anterior and posterior hippocampus; and anterior, posterior and middle cingulum. In addition, we evaluated the hypothesized ictal-onset zone (IOZ) for each participant who had seizures during their admission and sampled these areas for each participant during non-heatwave and heatwave epochs.

### Seizure identification and seizure counting

Evaluation was undertaken on the same days as were selected for analysis of IED discharges.

All clinical and subclinical seizures were manually counted over the 24-h period (including both wake and sleep stages) and verified by two experienced EEG readers (D.J.-J. and B.D.). Seizure identification followed published methodology.^23^ Seizures were classified according to international guidelines.^24^ All clinical and subclinical ictal activity longer than 10 s was considered relevant.^25^

### *Post hoc* analysis

After the initial hypothesis testing, we undertook a further analysis examining whether variations in IEDs and seizures might be related to the temperature *difference* between the selected non-heatwave and heatwave days. We calculated the percentage change in daily maximum temperature between the non-heatwave day and the heatwave days from which epochs were sampled. We then compared the percentage change in daily maximum temperature from non-heatwave to heatwave against the percentage change in number of IEDs across all the sampled depth electrode contacts per individual participant from non-heatwave to heatwave epochs. Additionally, we compared the percentage change in daily maximum temperature from the non-heatwave to heatwave day against the percentage change in number of seizures per individual participant from the non-heatwave day to the heatwave day.

### Statistical analysis

Statistical analysis was performed in GraphPad Prism (GraphPad Prism version 8.0.0, GraphPad Software, San Diego, CA, USA). We compared the total number of IEDs by sampled depth electrode contact for each individual participant over the total 40 min examined between non-heatwave and heatwave epochs using a Wilcoxon matched-pairs signed rank test to evaluate differences in overall IED numbers between the two epochs, using one tail as our hypothesis was that there would be more IEDs during the heatwave. We excluded electrodes from which no IEDs were recorded at all in either condition (heatwave and non-heatwave), as these electrodes are uninformative. Significance was set at *P* ≤ 0.05. We also compared the number of seizures during the selected non-heatwave and heatwave day using a one-tailed Wilcoxon matched-pairs signed rank test, comparing by participant. Significance was set at *P* ≤ 0.05.

For the *post hoc* analysis, we ranked the percentage increase in temperature between the non-heatwave and heatwave day from one (largest increase) to nine (smallest increase) for the nine participants. We also ranked the percentage increase in IED number between the non-heatwave and heatwave day from one (largest increase) to nine (smallest increase). We compared these rankings using a Spearman’s rank correlation coefficient (where 0 signifies no relationship). We used the same methodology to compare the rankings of percentage change in temperature between the non-heatwave and heatwave day with the percentage change in seizure frequency between the two days.

## Results

### Cohort

Eleven participants underwent icEEG during heatwaves between 01 May 2015 and 31 August 2022. Two participants were excluded: one participant was explanted on the first day of a heatwave at 0800 (outdoor temperature 23°C); the other participant was excluded from the study as no mesial temporal structures were sampled. The remaining nine participants included in the study (six men, three women) had a median age of 30 (range 24–39) years (see Table 1 for details). Non-heatwave 24-h peak temperature recordings ranged from 24.6 to 27.7°C. Heatwave 24-h peak temperatures ranged from 28.5 to 30.1°C. In four participants, non-heatwave epochs were sampled at least 48 h before the heatwave epochs, whilst in four participants, non-heatwave IED sampling was performed at least 48 h after the end of the heatwave. In one participant (#7), non-heatwave IED sampling was performed 24 h after the end of the heatwave. These variations were imposed by the timing of the heatwave with respect to the icEEG study and were not under our control.

**Table 1:** Participant demographics

Participant	Age	Sex	Habitual pre-admission seizure frequency	ASMs on admission	ASM level on the days of IED or seizure counting (compared to baseline pre-admission doses)	Non-heatwave day peak temperature	Heatwave day peak temperature	Final diagnosis after icEEG interpretation
1	30	M	1/week	CBZ TPM ZNS	100%	26.9	30.1	Right temporal lobe epilepsy
2	36	M	2–3/week	ESL PER	100%	25.8	28.9	Left temporal lobe epilepsy
3	39	F	daily	ZNS OXC LTG CLB	100%	24.6	28.5	Focal epilepsy, not localized
4	26	M	3–4/week	BRIV CBZ VPA	100%	24.7	29.7	Focal epilepsy, not localized
5	26	F	Several/week	CBZ LCM ZNS	0%	24.7	30	Focal epilepsy, not localized
6	24	M	1–2/week	LEV LTG CLB	0%	25.4	29.6	Right temporal lobe epilepsy
7	32	F	3–4/week	ESL LTG CLB	100%	26.7	28.6	Left temporo-occipital epilepsy
8	25	M	1–2/week	CLB LTG BRIV	100%	27.7	30	Right temporo-occipital epilepsy
9	39	M	1/week	PER	100%	27.3	29.8	Left temporal lobe epilepsy

Participants demographics, habitual seizure frequency, ASM at the time of admission to the unit, relative ASM level at time of IED counting (100%, full ASM doses; 0%, ASMs completely withheld), peak temperature on non-heatwave and heatwave day of IED counting are shown. CBZ, carbamazepine; TPM, topiramate; ZNS, zonisamide; ESL, eslicarbazepine; PER, perampanel; OXC, oxcarbazepine; LTG, lamotrigine; CLB, clobazam; VPA, valproate; LCM, lacosamide; BRIV, brivaracetam; IED, inter-ictal discharge.

### Genomics

For the eight participants with WGS data available (participant #5 did not have any genetic data available), no qualifying causal rare variants were found. This is in keeping with their clinical syndromes (Table 1) and indicates none had known genetically-driven temperature-sensitive epilepsies. The PRS for these eight participants are shown in Supplementary Fig. 2, with 7/8 falling above the interquartile range and participant #1 having the lowest PRS score for epilepsy in this group.

### IED numbers in heatwave and non-heatwave epochs

The depth EEG electrode contacts from which IEDs were sampled are listed in Supplementary Table  2.

**Figure 3:**
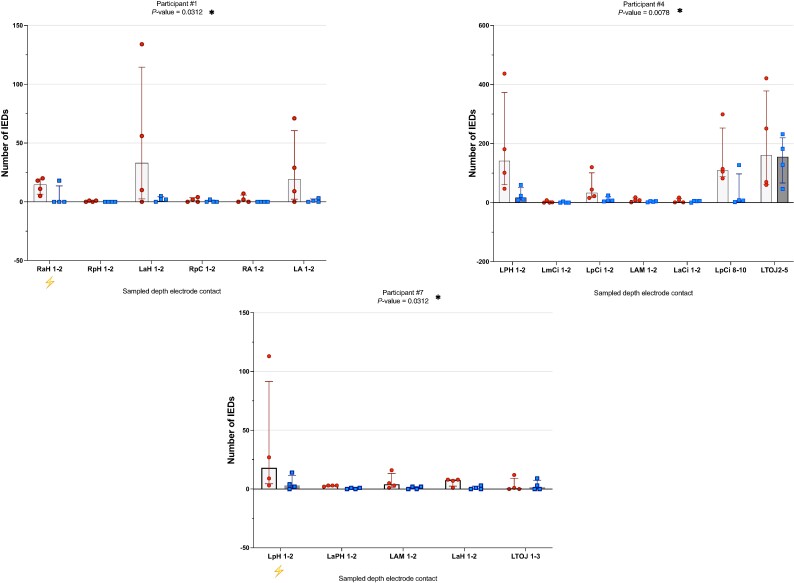
**Comparison of IED numbers per depth electrode contact per individual participant between non-heatwave and heatwave days; significant differences.** Number of IEDs (*y*-axis) per depth electrode contact (*x*-axis) during the two states, non-heatwave (dark bar, squares) and heatwave (light bar, circles), in the nine individual participants. Individual squares and circles within bars represent the IED count during the separate 10-min epochs. The top of each bar represents the median IED number per state and depth electrode contact. Bars are ordered chronologically depending on whether the heatwave or non-heatwave day occurred first. Depth electrode contacts with no sampled IEDs were omitted. The lightning bolt represents the area of the hypothesized IOZ. Please note that participant #4 was deemed non-localized (see Supplementary Table 2). A Wilcoxon matched-pairs signed rank test was used to evaluate the differences in overall IED numbers between the two epochs, using one tail. Significance was set at *P* ≤ 0.05. Stated depth electrode contact numbers correspond to the depth electrode contact sampled from. RaH, right anterior hippocampus; RpH, right posterior hippocampus; LaH, left anterior hippocampus; RpC, right posterior cingulum; RA, right amygdala; LA, left amygdala; LpH, left posterior hippocampus; LaCi, left anterior cingulum; LpCi, left posterior cingulum; LAM, left amygdala; RaCi, right anterior cingulum; RpCi, right posterior cingulum; Ram, right amygdala; LmCi, left middle cingulum; LTOJ, left temporo-occipital junction; LaPH, left anterior parahippocampal gyrus.

**Figure 4:**
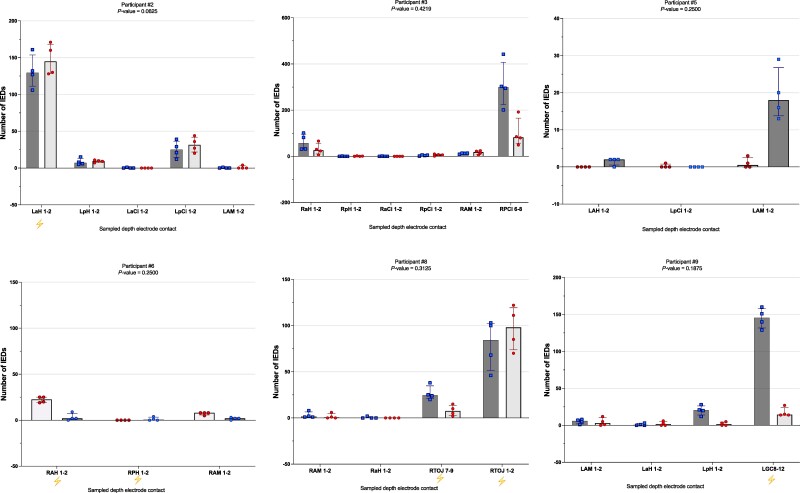
**Comparison of IED numbers per depth electrode contact per individual participant between non-heatwave and heatwave days; non-significant differences.** Number of IEDs (*y*-axis) per depth electrode contact (*x*-axis) during the two states, non-heatwave (dark bar, squares) and heatwave (light bar, circles), in the nine individual participants. Individual squares and circles within bars represent the IED count during the separate 10-min epochs. The top of each bar represents the median IED number per state and depth electrode contact. Bars are ordered chronologically depending on whether the heatwave or non-heatwave day occurred first. Depth electrode contacts with no sampled IEDs were omitted. The lightning bolt, if present, represents the area of the hypothesized IOZ. Please note that participants #3 and #5 were deemed non-localized (see Supplementary Table 2). A Wilcoxon matched-pairs signed rank test was used to evaluate the differences in overall IED numbers between the two epochs, using one tail. Significance was set at *P* ≤ 0.05. Stated depth electrode contact numbers correspond to the depth electrode contact sampled from. RaH, right anterior hippocampus; RpH, right posterior hippocampus; LaH, left anterior hippocampus; RpC, right posterior cingulum; RA, right amygdala; LA, left amygdala; LpH, left posterior hippocampus; LaCi, left anterior cingulum; LpCi, left posterior cingulum; LAM, left amygdala; RaCi, right anterior cingulum; RpCi, right posterior cingulum; Ram, right amygdala; LmCi, left middle cingulum; RTOJ, right temporo-occipital junction; LGC8, left inferior temporal gyrus.

In three participants (#1, #4 and #7; Fig. 3), there were significantly more IEDs over the 40 sampled minutes during heatwave epochs compared to the non-heatwave epochs (all three were on their full admission doses of ASMs during both epochs). No significant difference was seen for the other six participants (Fig. 4).

*Post hoc* analysis examining the percentage change in daily maximum temperature between the selected non-heatwave and the heatwave day for each individual participant against the percentage change in the total IED number across all sampled areas during the non-heatwave and heatwave days showed a weak, non-significant, negative association (Spearman’s rank = −0.28).

### Seizure numbers

We reviewed 432 h of EEG recordings from the nine participants on the same days used for IED analysis: in total, 216 h were during the non-heatwave epochs and 216 h were during the heatwave epochs. We identified 63 seizures: 8 seizures during non-heatwave epochs and 55 during heatwave epochs (Table 2). In five participants, there were more seizures during the heatwave, including for two (#1 and #4) of the three participants with significantly more IEDs during the heatwave epoch, with participant #4 experiencing the greatest increase in seizure frequency. Two participants (#3 and #8) had partial reductions in ASMs between the earlier non-heatwave study day and the subsequent heatwave study day (see Supplementary Tables 3 and 4 for details on ASMs and seizures).

**Table 2:** Seizures during the non-heatwave and heatwave days

Participant ID	Number of seizures (non-heatwave)	Seizure type (non-heatwave)	Number of seizures (heatwave)	Seizure type (heatwave)
1	0	NA	1	FM (1)
2	1	FM (1)	3	FM (3)
3	5	FM (5)	13	FM (11), FNM (1), SC (1)
4	1	FM (1)	35	FM (34), SC (1)
5	1	FM (1)	0	NA
6	0	NA	0	NA
7	0	NA	0	NA
8	0	NA	3	FM (2), FNM (1)
9	0	NA	0	NA
Total	8	NA	55	NA

Number of clinical and subclinical seizures and time (hours) in non-heatwave and heatwave conditions for each participant. Seizures were classified according to established international guidelines.^26^ FM, focal motor; SC, subclinical; FNM, focal non-motor; NA, not applicable.

**Figure 5:**
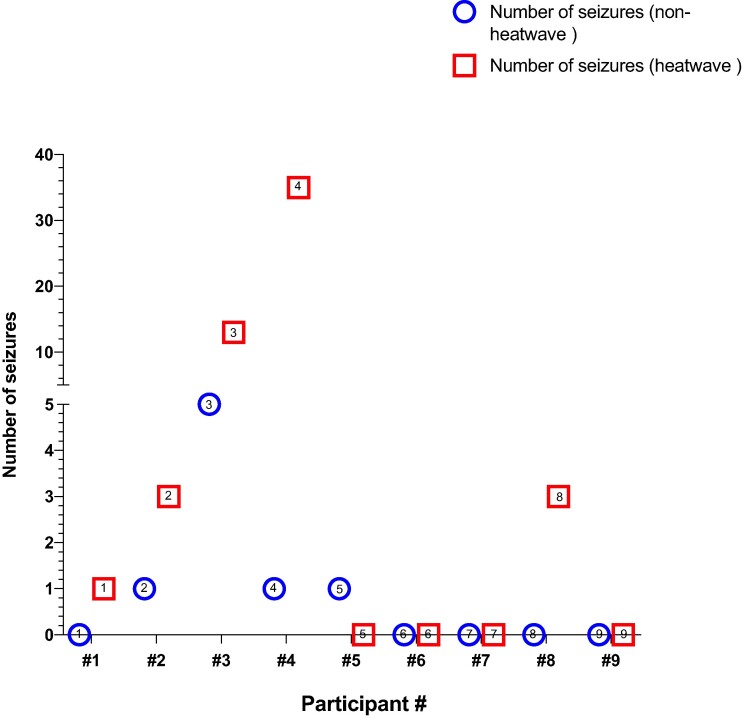
**Number of seizures for each participant included in the study on the non-heatwave and heatwave days.** Circles represent the number of seizures during the non-heatwave day. Squares represent the number of seizures during the heatwave day. Numbers in symbols are the participant #. Symbols are presented in chronological order of the day of seizure counting. There was a significant difference (*P* < 0.05) between the non-heatwave and heatwave days. A one-tailed Wilcoxon matched-pairs signed rank test was used, and significance was set at *P* ≤ 0.05.

One participant (#5) had a reduction in seizures in the heatwave, and three participants had no change in seizures (Fig. 5). There was a significant difference (*P* = 0.047) when comparing the number of seizures by individual participant between the non-heatwave and heatwave day across the group as a whole. For analysis of seizure numbers, because this is a brain systems phenomenon, we included all participants, even those who had no seizures during heatwave or non-heatwave days; as a sensitivity analysis, exclusion of participants (#6, #7 and #9) who had no seizures during either epoch did not alter the statistical significance of our findings (refer to Supplementary Table 4 for seizure details with respect to study days).

Finally, in the *post hoc* analysis, there was no correlation (Spearman’s rank = 0) for the comparison between the percentage increase in daily maximum temperature between the non-heatwave and the heatwave days against the percentage increase in seizures during the selected non-heatwave and heatwave days.

## Discussion

We explored the impact of temperature on brain excitability using two measures, IED and seizure number, in a cohort of individuals with known genomic background, to test the hypothesis that higher ambient temperatures increase brain excitability, based on the known sensitivity of ion channels to ambient temperature, in the context of global climate change and its potential effects on the human brain. We show that IEDs can increase in relation to heatwaves as recorded by depth electrodes in people undergoing evaluation for possible surgical treatment of their epilepsy. In three individuals, the number of IEDs increased in the heatwave epoch; in six individuals, there was no significant change between epochs. For the other measure of brain excitability we tested, seizure frequency, we observed an increase in seizure number during heatwaves across the participants as a group. None of the participants had a known genetic epilepsy as determined both by the clinical diagnosis and evaluation of the whole-genome data. However, all had PRSs for epilepsy at the higher end of the range presented from a large group of people with epilepsy, indicating an elevated overall background common variation risk for epilepsy in keeping with the serious nature of the epilepsy in these individuals, with no obvious difference between individual participants (Supplementary Fig. 2). Overall, this study supports the assertion that ambient temperature influences brain excitability and indicates the need for additional research in this important area. Of particular note, none of the participants had a known genetic epilepsy, yet 3/9 showed a significant increase in IED frequency during heatwave epochs compared to non-heatwaves epochs, and as a group, there was a marginally significant increase in seizure frequency during heatwave compared to non-heatwave epochs, which suggests that it is not only people with (typically rare) genetic epilepsies who may be particularly vulnerable to the impacts of heatwaves. These findings raise concerns about the impact of heatwaves for people with a range of different types of severe epilepsies even when these are not obviously due to a monogenic cause.

Populations distributed across latitudes show genetic adaptation to historical local climate conditions.^26-28^ Moreover, at the individual level, some individuals relish hotter days, whilst others find them more challenging, the basis for which remains unclear, but with contributions postulated from genetic factors.^29,30^ All the individuals in our study, except participant #9, are of European ancestry, and therefore in general likely adapted genetically to temperatures typically below the ranges to which they were exposed during the heatwaves included in this study, but inter-individual differences to heat tolerance are important and may exceed ethnic differences.^29^ Participant #9 is of admixed American ancestry on genetic grounds: this participant did not show any differences in IEDs or seizure numbers between the heatwave and non-heatwave epochs, but we cannot draw any more conclusions from data from one individual alone. Individual thermal comfort and temperature preferences were not documented at the time of icEEG recording: it remains possible these factors influenced the observed differences in IED and seizure numbers between heatwave and non-heatwave epochs. Our study demonstrates the complexity of evaluating heat effects on human brain function: adaptations to preserve healthy brain function in worsening heatwaves expected with climate change will need to account for such complexities and will need systems approaches.

There is a lack of data on the most biologically relevant way to quantify temperature changes that may affect human brain health. Published data on climate change show extensive disparities in study parameters and reporting, with associations reported between worsening disease parameters (e.g. stroke incidence; admissions for particular conditions) and a variety of different measures of weather, such as diurnal or nocturnal peak temperatures, diurnal temperature excursions, standardized deviation from seasonal norms or the effect of cumulative or lagged (i.e. specified intervals between weather events and the disease parameter in question) measures of temperature. Here, we chose to study peak temperature observed over a 24 h period and sought to ensure a minimum interval of 48 h between these epochs (achieved for 8/9 participants). Our *post hoc* analysis was undertaken to evaluate the data using a second approach (percentage change in temperature versus percentage change in measure of brain excitability): the analysis did not show any significant association. We also acknowledge that the thermal mass of the building itself, metabolic activity, clothing and ventilation levels will have had effects on the indoor temperature experienced and that lagged effects may also have been important. However, by capturing both epochs during the same episode of icEEG recording and by imposing several other conditions on the selection of the studied intervals, we aimed to minimize these potential influences on IED and seizure occurrences. We acknowledge that for two participants, there was partial reduction in ASM doses after the non-heatwave day and before the heatwave day (#3 and #8, as detailed in Supplementary Table 3), although both were on full medications on the day of the heatwave. We consider it unlikely that this was material to our findings, noting for example that there was no change in inter-ictal findings between these days for these participants and the overall patterns of seizures by participant (Supplementary Table 4). An important strength of our study is that the effect of indoor temperatures, albeit interpolated, is considered: the vast majority of published studies examining the links between temperature and human health use external temperatures, whilst in high-income countries, most people spend more than 90% of their lives indoors. Our study therefore provides information that is more likely to be relevant to human health, at least in high-income countries.

Intracranial EEG offers the unique advantage over scalp EEG that longer recordings are commonly undertaken and more tolerable and unaffected by scalp perspiration. However, our approach of using icEEG also imposes some limitations. The sample size was small: icEEG recordings are only appropriate in carefully selected people with epilepsy who may not be representative of most people with epilepsy. Despite the increasing occurrence of heatwaves in the UK, periods of warm spells occurred for an average length of only 13 days a year from 2008 to 2017,^16^ limiting the number of eligible studies. IcEEG is only required in few epilepsy surgery candidates, and given its invasiveness and need for input from a range of different medical specialities, only a limited number are performed.^31^ Other long-term EEG methodologies, such as those employing chronic ambulatory icEEG recording, are being developed. One study using an implanted responsive neurostimulation system showed phase locking of seizure timing with a range of monitored personal variables, including skin temperature,^32^ which itself has a complex relationship to ambient temperature. We relied on modelled data prior to 2022 as we did not have indoor recordings from the telemetry unit until 29 April 2022. We are now monitoring prospectively both outdoor and indoor temperatures. Sleep deprivation can promote IEDs and seizures in people with epilepsy.^33^ Heatwaves can disrupt sleep patterns, causing fatigue and sleep deprivation.^5,34^ We did not evaluate how well the participants slept during their time in hospital. These are mechanistic details requiring prospective work and do not detract from the observed association between higher ambient temperature and brain excitability. In addition, we focused on awake EEG epochs for analysis. It is challenging to distinguish wakefulness from drowsiness and N1 sleep on icEEG recordings even for the trained observer, but we did exclude deep and rapid eye movement (REM) sleep.^35^ Using comparable participant states between the non-heatwave and heatwave days was a strength of the study.^36,37^ IED counting was performed manually. We see this as a strength of the study: whilst it is time-consuming and prohibits evaluation across longer time frames, expert manual detection avoids inclusion of false positives, a vulnerability of automated IED detection software.^38^

There are few explorations published about the potential impacts of climate change on the human brain and, specifically, on the human brain in disease.^39,40^ People with neurological diseases, their carers and clinicians are concerned about climate change.^41^ The Earth has already passed, at least temporarily, the 1.5°C threshold set by the 2015 Paris Agreement far sooner than predicted. Our study raises important concerns about brain excitability during heatwaves. We urgently need more data to protect people with neurological diseases and ensure the best health outcomes from adaptation and mitigation actions being taken against climate change.

## Supplementary Material

fcae269_Supplementary_Data

## Data Availability

Summary data used for the analyses are available, under appropriate data sharing agreements subject to our ethics protocol, for bona fide researchers. Individual-level data are not available due to the risk of de-identification. Research on the de-identified patient data and control data used in this publication can be carried out in the Genomics England Research Environment subject to a collaborative agreement that adheres to patient-led governance. All interested readers will be able to access the data in the same manner that the authors accessed the data. For more information about accessing the data, interested readers may contact research-network@genomicsengland.co.uk or access the relevant information on the Genomics England website: https://www.genomicsengland.co.uk/research.

## Code availability

No bespoke code was used for this study. All code used in the manuscript is in the public domain already and has been appropriately referenced. We have added an additional reference to the R package *Ranger*, which was used to implement the random forest model in RStudio, allowing readers to replicate the analysis with their own data.
