# Supplementary material for: The influence of temperature and genomic variation on intracranial EEG measures in people with epilepsy
Source: Brain Commun. 2024 Sep 10;6(5):fcae269. doi: 10.1093/braincomms/fcae269 (PMC11383581; doi:10.1093/braincomms/fcae269)
Supplement: fcae269_Supplementary_Data [file fcae269_supplementary_data.pdf]

Supplementary Materials

Genomic Analysis

All low Genotype Quality (GQ) calls (GQ values < 20) and calls with low read depth (DP, DP < 10) were excluded from downstream analyses. All 520 epilepsy-related genes (monoallelic, biallelic and X-linked) categorised as "green" (diagnostic level of evidence) genes in the 'Early-onset or Syndromic Epilepsy' gene panel (Version 4.0, March 22, 2023), developed by Genomics England, were used for rare variant analysis.[1] Variants across these genes were extracted from variant call format files (VCF files) of each sample and annotated using VEP.[2] All exonic variants that could modify the amino acid sequence and splice site variants with gnomAD minor allele frequency ≤0.0005 (gnomAD whole-genome version 3.1.2) were considered as qualifying variants. For one participant, WGS was performed through the Genomics England UK 100,000 Genomes Project (GEL) (The National Genomic Research Library v5.1, Genomics England. https://doi.org/10.6084/m9.figshare.4530893.v7): these variants were annotated using VEP with the same genotype quality and minor allele frequency thresholds. The pathogenicity of all the qualifying variants was evaluated according to the American College of Medical Genetics and Genomics Association (ACMG) guidelines.[3]

PRS for epilepsy were calculated in eight of the nine participants in this study, GEL Epilepsy and GEL Control cohorts. The GEL Epilepsy cohort comprised 2,782 adults with epilepsy, while the GEL Control cohort we constructed comprised 1,157 unaffected participants. PRS for epilepsy were estimated using genome-wide association study summary statistics generated by the International League Against Epilepsy (ILAE) Consortium on Complex Epilepsies.[4] After applying quality control steps, we computed PRS based on the overlap of the remaining quality-controlled single-nucleotide polymorphism in the study groups, as previously described.[5] PRS for each individual were obtained using the clumping and thresholding method implemented by PRSice-v2.3.3 across a set of P-value thresholds (PT= 10^{-4}, 10^{-3}, 10^{-2}, $5x10^{-2}$, 10^{-1}, 0.5, 1).[6] The PT that best fit the target trait across the thresholds was identified as in our previous work (Supplementary Figure 1).[5]

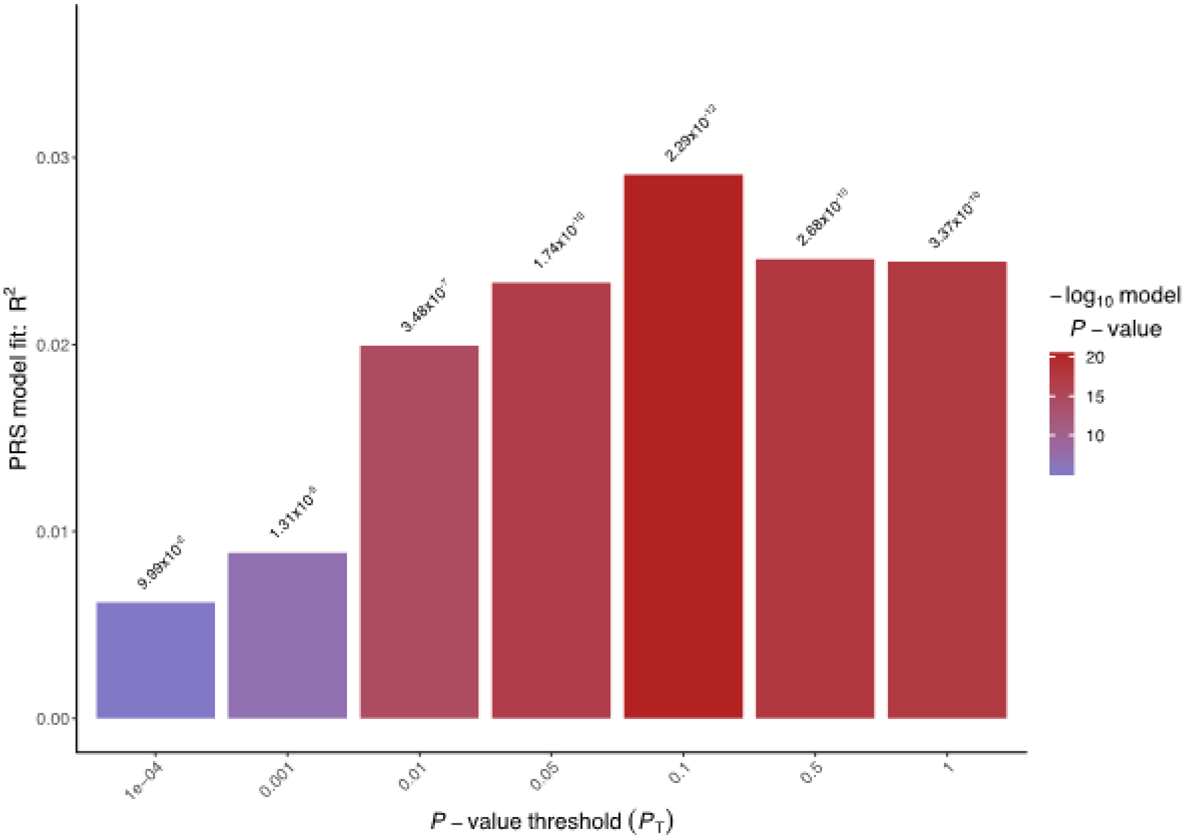

Supplementary Figure 1. P-value thresholding for polygenic risk score (PRS) analysis.

Bar plot displaying the model fit of the epilepsy PRS at the stated P-value threshold (GEL Epilepsy vs GEL Controls): The model fit of the epilepsy PRS shows the best predicting P-value threshold at 10^{-1}. The epilepsy PRS explained around 3% (R^2=0.030) of the total phenotypic variance in the entire GEL epilepsy cohort. P-values were calculated to assess statistical significance using a t-test.

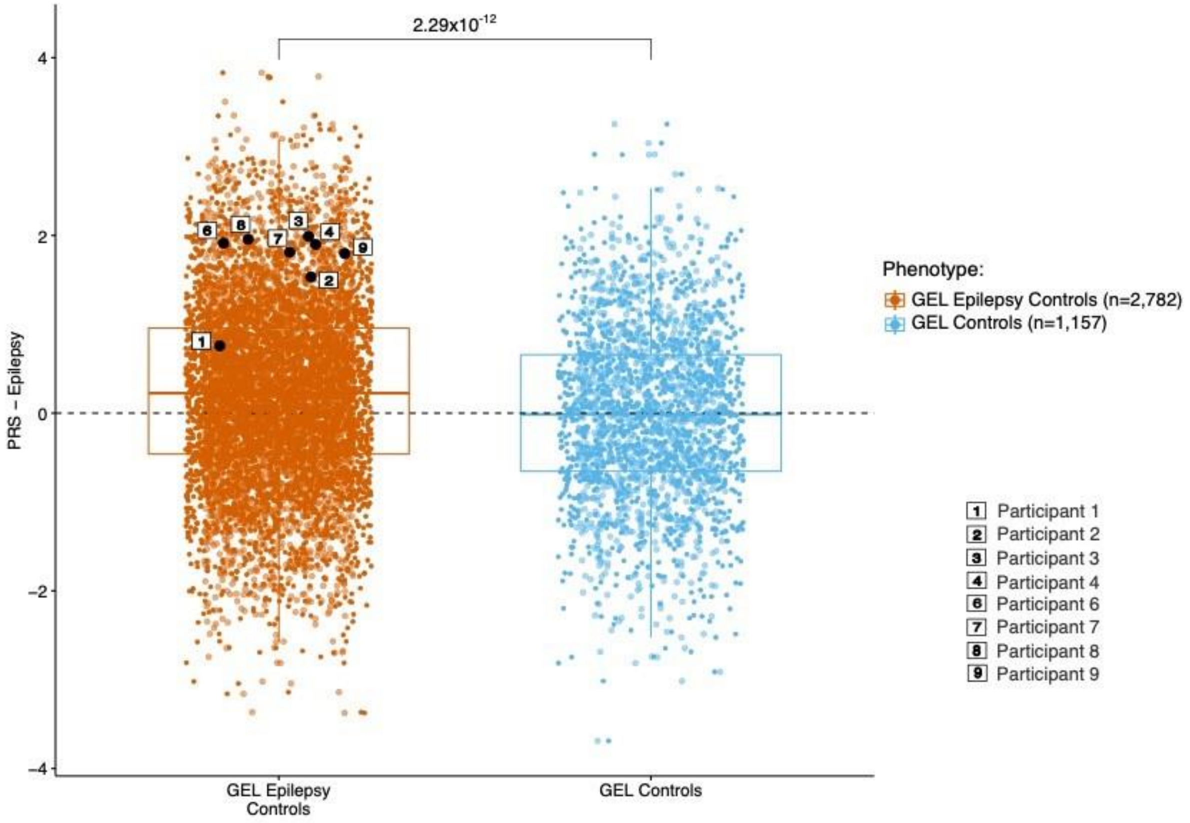

Supplementary Figure 2. Polygenic risk scores for epilepsy.

The polygenic risk scores (PRS) for eight participants who had whole genome sequencing (WGS) data available (participant #5 did not have any genetic data available) compared with control participants from GEL (best fit P-value threshold equal to 10^{-1}, see Supplementary Figure 1). The GEL Epilepsy cohort comprised 2,782 adults with epilepsy and the GEL Control cohort comprised 1,157 unaffected participants. Seven out of the eight fall above the interquartile range, with participants #1 and #2 having the lowest PRS for epilepsy of this group. (GEL = Genomics England 100,000 Genomes Project, PRS = Polygenic Risk Scores). P-values were calculated to assess statistical significance using a t-test.

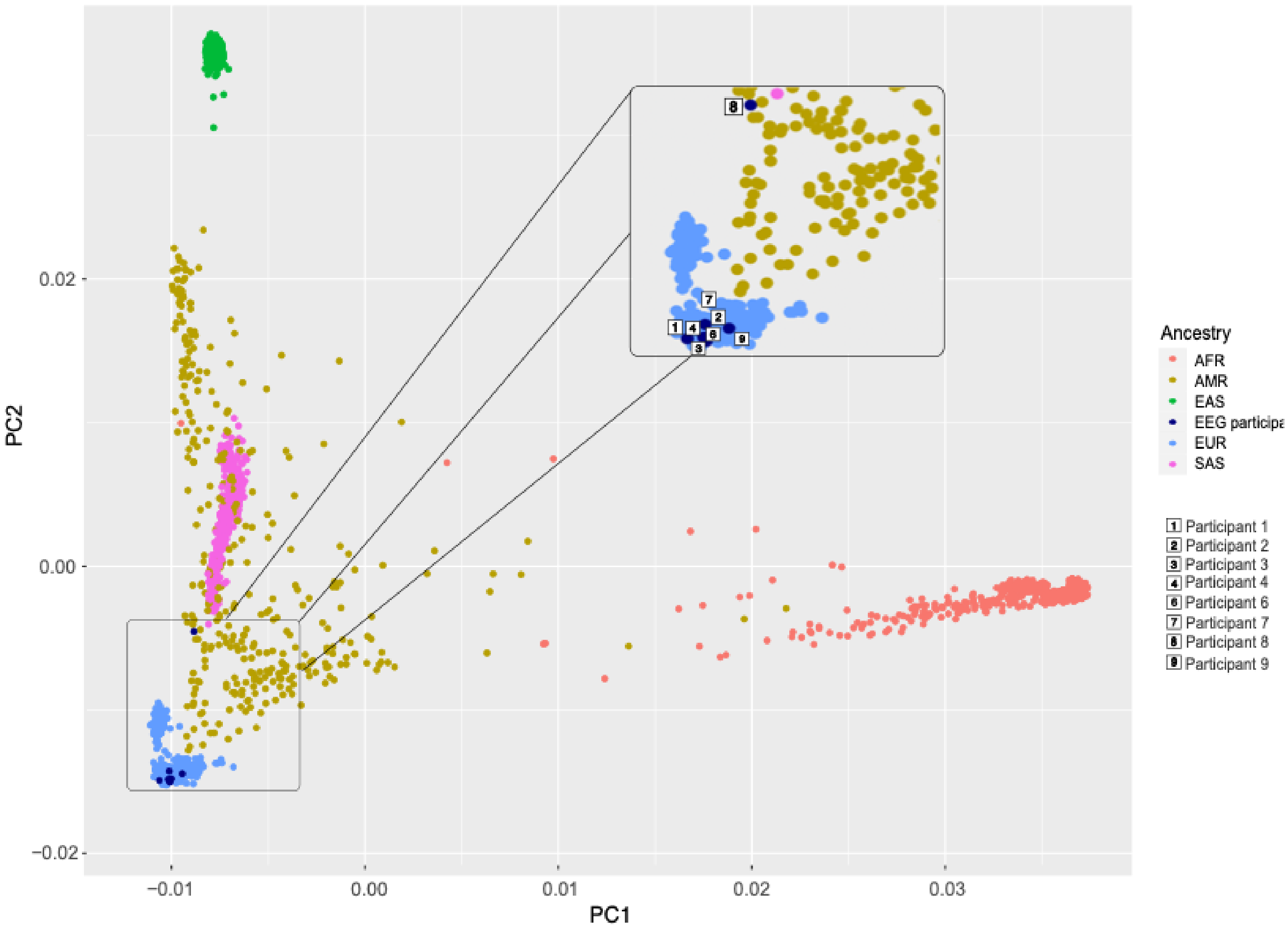

Supplementary Figure 3: Ancestry of the individuals in the study cohort determined by comparison with the 1000 Genomes Project reference dataset. A principal component 1 (PC1) vs principal component 2 (PC2) plot was used to visualise sample ancestry.

AFR: 1000 Genomes African samples; AMR: 1000 Genomes admixed American samples; EAS: 1000 Genomes East Asian samples; EUR: 1000 Genomes European samples; SAS: 1000 GenomesSouth Asian samples.

EEG participants: Eight participants underwent icEEG during heatwaves between 01.05.2015 and 31.08.2022, and had available DN

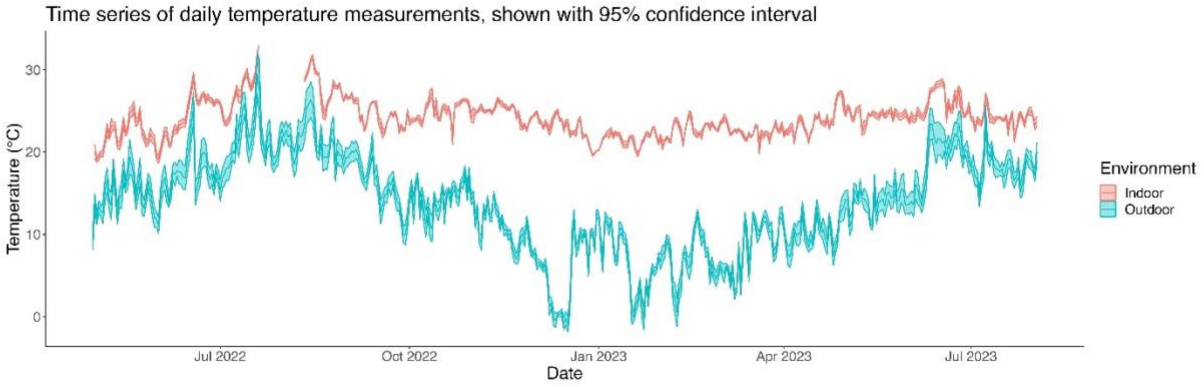

Supplementary Figure 4. Time-series plot of the indoor and outdoor temperature data aggregated to daily means. The figure is comprised of *n*=450 daily estimates of the 24-hour-mean temperature for each environment (indoor and outdoor). Indoor temperature data was collected at sub-hourly intervals from the telemetry unit, National Hospital for Neurology and Neurosurgery. Outdoor temperature was provided by the VisualCrossing API at St. James Park, London, monitoring station. Note that indoor data were missing between the dates of 20.07.2022-09.08.2022 as the monitor memory was full. During the summer months (June-August) the telemetry unit experienced a temperature rise in line with the rising external temperatures. Jul=July, Oct=October, Jan=January, Apr=April.

Random Forest Model

A random forest is a machine learning method which utilises ensemble techniques, where a number of subsets of the original dataset are created, known as bagging. A prediction is computed for each subset of the original dataset and an average of the models created from each subset computed to produce a combined or "overall" prediction using a flowchart-like tree structure. The outdoor meteorological variables included in the final model were: outdoor temperature (°C), outdoor relative humidity (%), outdoor wind speed (kph) and outdoor solar radiation (W/m^2), all available from the Visual Crossings API at the St James Park location. The month of the year was additionally included to account for seasonal variations in indoor temperature, such as space-heating.

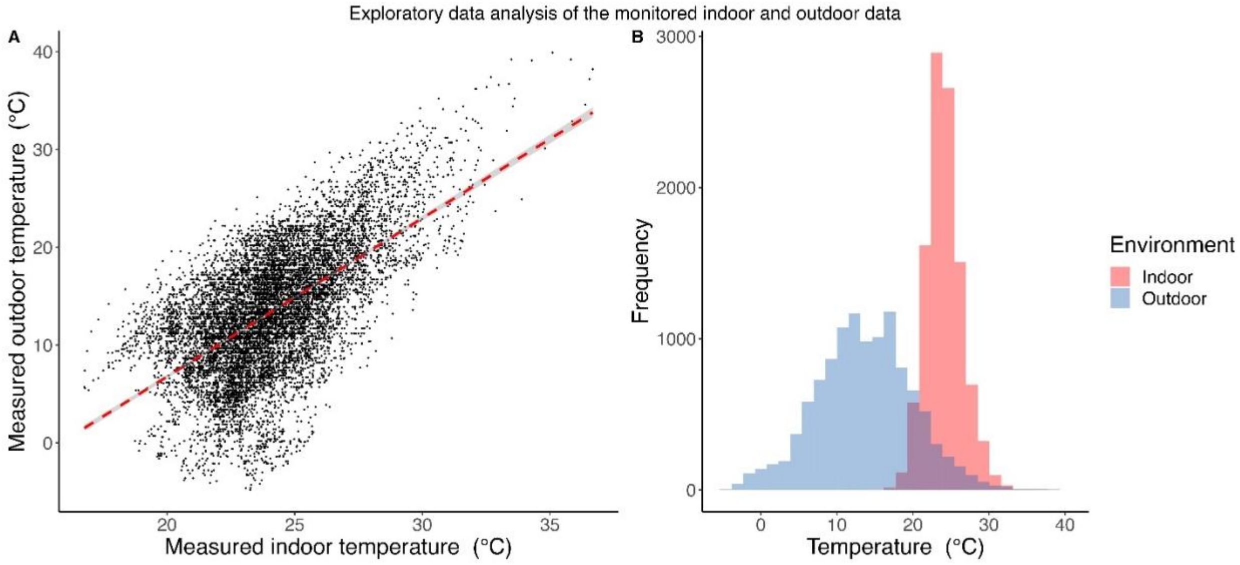

Supplementary Figure 5. Measured indoor and outdoor data at NHNN.

Scatterplot of measured indoor and outdoor data (A) and overlaid histograms showing the temperature distribution of measured data in the indoor and outdoor environment at the telemetry unit, NHNN, Queen Square, London WC1N 3BG (B). Indoor temperature data were collected at sub-hourly intervals from the telemetry unit, NHNN. Outdoor temperature was provided by the MetOffice Had UK-Grid Gridded Climate Observations at the location of the telemetry unit, NHNN. Data consists of monitored data at hourly time-steps.

To assess the model's goodness-of-fit, the predictions from the testing dataset were subtracted from the real-life indoor measurements, with results shown in Supplementary Table 1. On average, the model showed minimal variation between actual and predicted values.

Supplementary Table 1. Summary of goodness-of-fit between (actual – predicted) values for indoor temperature and relative humidity.

	Min.	25th quantile	Median	Mean	75th quantile	Max.
Indoor temperature (°C)	-3.19	-0.22	0.00	0.00	0.23	2.56

Finally, actual and predicted values were mapped against each other and an R^2 computed for each month of data. The results for indoor temperature are shown in Supplementary Figure 6.

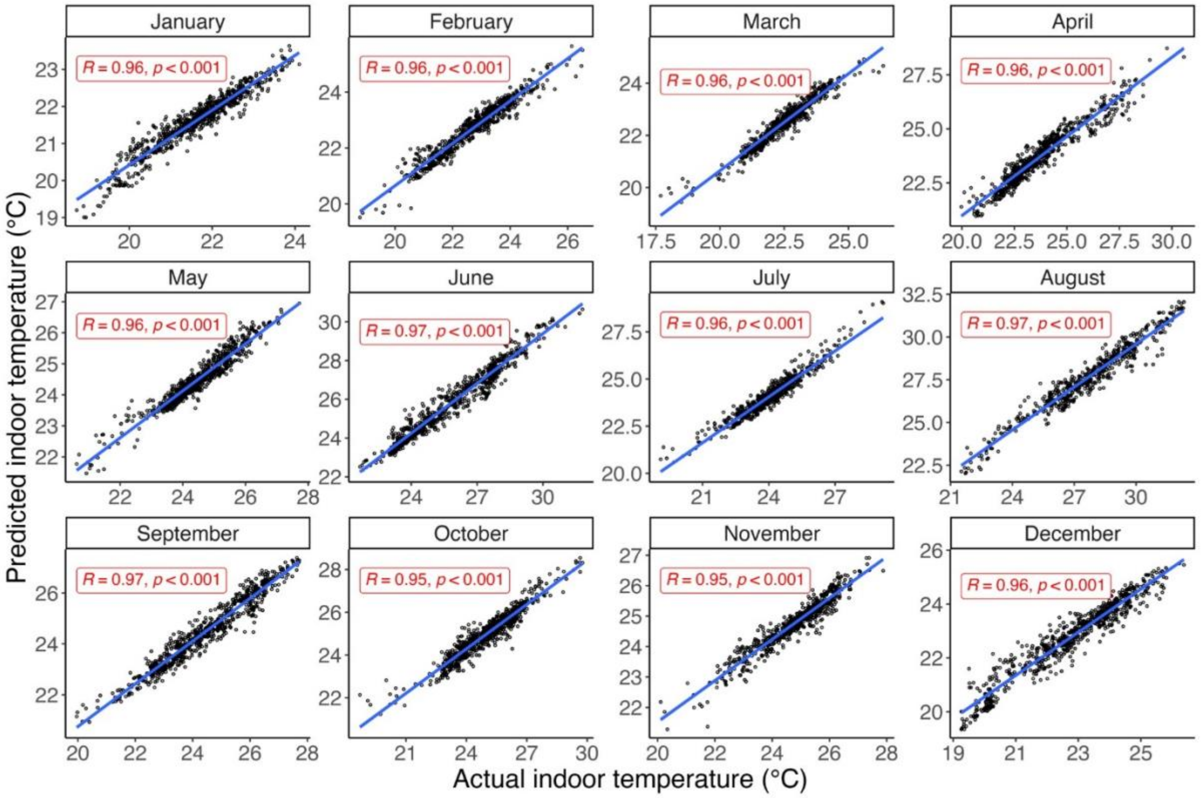

Supplementary Figure 6. Actual versus predicted indoor temperature estimates. Actual versus predicted indoor temperature estimates for each month, shown with the month-specific Pearsons correlation coefficient (*R*) and *p*-value. There were approximately ~744 data points for each month (24 hourly estimates, with differences due the varying number of days in each month), although August had fewer estimates (*n*= 559*)* due to missing data points. All correlations were significant at the *p*=0.001 level of significance.

Supplementary Table 2. Implanted depth electrode contacts for IEDs identification.

RaH: right anterior hippocampus; RpH: right posterior hippocampus; LaH: left anterior hippocampus, LpH: left posterior hippocampus; RaCi: right anterior cingulum; RpCi: right posterior cingulum; LaCi: left anterior cingulum; LmCi: left middle cingulum; Ram: right amygdala, Lam: left amygdala, LTOJ: left temporo-occipital junction; RTOJ: right temporo-occipital junction; LpPH: left posterior parahippocampal gyrus.

Participant ID	Hippocampus	Cingulate	Amygdala	Additional electrodes	Ictal onset zone
**#1**	RaH, RpH, LaH	RpCi	Ram, Lam		Right hippocampus
**#2**	LaH, LpH	LaCi, LpCi	Lam		Left hippocampus
**#3**	RaH, RpH	RaCi, RpCi	Ram	Supramarginal gyrus	Non-localised
**#4**	LpH	LaCi, LmCi, LpCi	Lam	Supramarginal gyrus, LTOJ	Non-localised
**#5**	LaH	LpCi	Lam		Non-localised
**#6**	RaH, RpH	None	Ram		Right mesial temporal lobe
**#7**	LpH, LaH, LaPH	LpCi	Lam	LTOJ	Left posterior parahippocampal gyrus/lingual gyrus
**#8**	RaH	None	Ram	RTOJ	Right temporo-occipital junction
**#9**	LaH, LpH	None	Lam	Inferior temporal gyrus	Inferior temporal gyrus

Supplementary Table 3. Details of antiseizure medication reductions and timing with respect to study days and day of intracranial EEG recording.
ASM: anti-seizure medication.

Participant ID	Changes in IEDs/ seizures on heatwave day compared to non-heatwave day	Total days of intracranial EEG recording	Day of admission selected as non-heatwave day	Non-heatwave day ASM dose (% of baseline total)	Day of admission selected as heatwave day	Heatwave ASM dose (% of baseline total)	Anti-seizure medication (ASM) reduction details
**#1**	yes (increased)/ yes (increased)	11	7	100	2	100	100% throughout the whole study; no ASM reduction.
**#2**	no/yes (increased)	9	3	100	5	100	100% throughout the whole study; no ASM reduction.
**#3**	no/yes (increased)	14	5	100	10	100	Zonisamide maintained at 100% throughout admission. Oxcarbazepine reduced to 50% on evening of day 6/14, reinstated to 100% on evening of day 9/14. Lamotrigine reduced to 50% on evening of day 6/14, reinstated to 100% on evening of day 9/14.
**#4**	yes(increased)/ yes(increased)	8	4	100	2	100	100% throughout the whole study; no ASM reduction.
**#5**	no/yes (decreased)	19	17	0	14	0	Several days at 0% in both conditions<br>ASM at 0% from day 10/19. Reinstated on day 18/19.
**#6**	no/no	22	8	0	12	0	25% on the day before both conditions (non-heatwave and heatwave); fluctuating ASM reduction – reduced to 25% on 6/22, 7/22, 0% on day 8/22, 25% 9/22, 0/% 10/22, 25% 11/22, 0% 12/22.
**#7**	yes(increased)/ no	14	3	100	2	100	No ASM reduction was implemented at the time of sampling – 100% until day 4 of recording, after both sampled days.
**#8**	no/yes (increased)	11	6	100	9	100	Clobazam maintained on 100% for the whole admission. Lamotrigine reduced to 50% on evening of day 7/11, increased to 100% on morning of day 9/11. Brivaracetam reduced to 50% on evening of day 7/11, increased to 100% on morning of day 9/11.
**#9**	no/no	10	3	100	9	100	ASM reduction from day 5/10 – 8/10, reinstated on day 8/10.

Supplementary Table 4. Details of antiseizure medication reductions and number of seizures (clinical and subclinical) with respect to study days and day of intracranial EEG recording.

ASM: anti-seizure medication; CS: clinical seizure; SC: subclinical seizure.
Day of recording selected as non-heatwave is in blue underlined text; day of recording selected as heatwave is in red bold text. Further days in the heatwave are in red.

Participant ID	Total days of intracranial EEG recording	Day of recording selected as non-heatwave day	Non-heatwave day ASM dose (% of baseline total)	Day of recording admission selected as heatwave day	Heatwave ASM dose (% of baseline total)	Seizure details<br>D = day<br>CS = clinical seizure<br>SC = subclinical seizure
#1	11	7	100	2	100	D1 = 0<br>**D2 = 1x CS; 1x SC**<br>D3 = 9x SC<br>D4 = 1x CS; 2x SC<br>D5 = 0<br>D6 = 1x SC<br>D7 = 0<br>D8 = 2x SC<br>D9 = 1x CS<br>D10 = 3x CS<br>D11 = 1x SC
#2	9	3	100	5	100	D1 = 0<br>D2 = 0<br>D3 = 1x CS<br>D4 = 0<br>**D5 = 2x CS**<br>D6 = 0<br>D7 = 2x CS<br>D8 = 1x CS<br>D9= 0
#3	14	5	100	10	100	D1 = 0<br>D2 = 0<br>D3 = 0<br>D4 = 1x CS; 1x SC<br>D5 = 5x CS<br>D6 = 1x CS; 1x SC<br>D7 = 3x CS; 2x SC<br>D8 = 29x CS; 6x SC<br>D9 = 12x CS<br>**D10 = 12x CS; 1x SC**<br>D11 = 0<br>D12 = 7x CS<br>D13 = 0<br>D14 = 0
#4	8	4	100	2	100	D1 = 1x CS<br>**D2 = 34x CS; 1x SC**<br>D3 = 3x CS<br>D4 = 1x CS<br>D5 = 1x CS<br>D6 = 0<br>D7 =0<br>D8 = 1x CS
#5	19	17	0	14	0	D1 = 0<br>D2 = 0<br>D3 = 0<br>D4 = 0<br>D5 = 0<br>D6 = 0<br>D7 = 0<br>D8 = 0<br>D9 = 0<br>D10 = 0<br>D11 = 0<br>D12 = 0<br>D13 = 0<br>**D14 = 0**<br>D15 = 0

						D16 = 2x CS<br>D17 = 1x CS<br>D18 = 0<br>D19 = 0
**#6**	22	8	0	12	0	D1 = 0<br>D2 = 0<br>D3 = 0<br>D4 = 0<br>D5 = 0<br>D6 = 0<br>D7 = 0<br>D8 = 0<br>D9 = 0<br>D10 = 0<br>D11 = 0<br>**D12 = 0**<br>D13 = 0<br>D14 = 0<br>D15 = 0<br>D16 = 0<br>D17 = 0<br>D18 = 0<br>D19 = 3x CS<br>D20 = 0<br>D21 = 0<br>D22 = 0
**#7**	14	3	100	2	100	D1 = 0<br>**D2 = 0**<br>D3 = 0<br>D4 = 0<br>D5 = 0<br>D6 = 0<br>D7 = 0<br>D8 = 0<br>D9 = 1x CS<br>D10 = 0<br>D11 = 0<br>D12 = 0<br>D13 = 1x CS<br>D14 = 0
**#8**	11	6	100	9	100	D1 = 0<br>D2 = 0<br>D3 = 0<br>D4 = 3x CS<br>D5 = 1x CS<br>D6 = 0<br>D7 = 0<br>D8 = 1x CS<br>**D9** = 3x CS<br>D10 = 1x CS<br>D11 = 0
**#9**	10	3	100	9	100	D1 = 0<br>D2 = 0<br>D3 = 0<br>D4 = 0<br>D5 = 0<br>D6 = 0<br>D7 = 4x CS<br>D8 = 5x CS; 14x SC<br>**D9 = 0**<br>D10 = 0

Supplementary references

1. Martin AR, Williams E, Foulger RE, et al. PanelApp crowdsources expert knowledge to establish consensus diagnostic gene panels. *Nat Genet*. 2019;51(11):1560-1565. doi:10.1038/s41588-019-0528-2
2. McLaren W, Gil L, Hunt SE, et al. The Ensembl Variant Effect Predictor. *Genome Biol*. 2016;17(1):122. doi:10.1186/s13059-016-0974-4
3. Richards S, Aziz N, Bale S, et al. Standards and guidelines for the interpretation of sequence variants: a joint consensus recommendation of the American College of Medical Genetics and Genomics and the Association for Molecular Pathology. *Genet Med Off J Am Coll Med Genet*. 2015;17(5):405-424. doi:10.1038/gim.2015.30
4. Stevelink R, Campbell C, Chen S, et al. GWAS meta-analysis of over 29,000 people with epilepsy identifies 26 risk loci and subtype-specific genetic architecture. *Nat Genet*. 2023;55(9):1471-1482. doi:10.1038/s41588-023-01485-w
5. Martins Custodio H, Clayton LM, Bellampalli R, et al. Widespread genomic influences on phenotype in Dravet syndrome, a “monogenic” condition. *Brain*. 2023;146(9):3885-3897. doi:10.1093/brain/awad111
6. Choi SW, O’Reilly PF. PRSice-2: Polygenic Risk Score software for biobank-scale data. *Gigascience*. 2019;8(7). doi:10.1093/gigascience/giz082
